# A study of pyrimidine base damage in relation to oxidative stress and cancer

**DOI:** 10.1038/sj.bjc.6605176

**Published:** 2009-07-14

**Authors:** H Iijima, H B Patrzyc, E E Budzinski, H G Freund, J B Dawidzik, K J Rodabaugh, H C Box

**Affiliations:** 1 Department of Cell Stress Biology, Roswell Park Cancer Institute, Elm and Carlton Streets, Buffalo, NY, 14263, USA; 2 Eppley Cancer Center, University of Nebraska, Omaha, NE, 68198-3255, USA

**Keywords:** ovarian cancer, DNA damage, oxidative stress, pyrimidine base damage

## Abstract

**Background:**

A long-standing hypothesis is that oxidative stress is a risk factor for cancer. Support for this hypothesis comes from observations of higher levels of oxidative damage in the DNA of WBC of cancer patients compared with healthy controls.

**Methods:**

Two generally overlooked types of DNA damage, the formamide modification and the thymine glycol modification, both derived from pyrimidine bases, were assayed as markers of oxidative stress. Damage levels were measured in the DNA of WBC of ovarian cancer patients and of healthy controls.

**Results:**

The levels of both modifications were higher in ovarian cancer patients than in healthy controls although in the case of the formamide modification age could not be ruled out as a factor.

**Conclusion:**

Our results in combination with other published measurements of oxidative DNA damage support the hypothesis that oxidative damage, on average, is higher in WBC of cancer patients than in healthy controls.

Oxidative stress in cells arises from an imbalance between pro- and antioxidants, in favour of the former. All cells exhibit a measurable level of oxidative DNA damage (Ames *et al*, 1992). Individuals exhibiting a higher level of oxidatively induced DNA damage than healthy controls may have an inherent deficiency either in DNA repair capacity or in protection against oxidative stress. Either deficiency implies a higher mutation rate and consequently a greater risk of cancer. Experimental data suggest that oxidatively induced DNA damage is, in fact, elevated in cancer patients.

Our study was based on measurements of pyrimidine base damage produced by reactive oxygen species (ROS) in white blood cells (WBC) from human blood samples. The base modifications of interest were the thymine glycol modification of thymine and the formamide modification derived from the breakdown of pyrimidine bases (Bailey *et al*, 2006; Greene *et al*, 2007). These modifications were examined in two aspects: (1) Production *in vitro* by radiation, both ionising and UVC; (2) The relationship of DNA base damage to cancer, particularly ovarian cancer.

## Materials and methods

Blood samples were obtained from ovarian cancer patients and from healthy volunteers. The case group was composed of successive patients admitted to the service having been diagnosed with stage 3 ovarian cancer. The control group, composed of healthy adult women, was randomly recruited from volunteer donors. The samples from patients were obtained after surgery and diagnosis, but before chemotherapy. Samples from patients and volunteer donors were obtained under Institute Review Board-approved protocols. Patients and volunteers were asked to fill out a questionnaire regarding medical and family histories and lifestyle. Information with respect to age proved to be especially important for this study.

**Figure 1:**
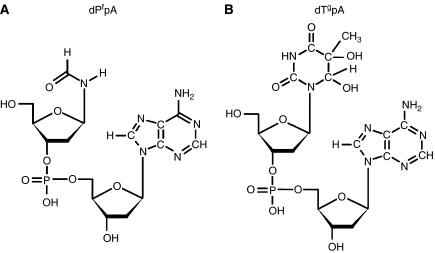
Forms of the formamide (**A**) and thymine glycol (**B**) modifications, measured by LC-MS/MS.

Buffy coat was isolated by centrifugation from fresh blood collected with EDTA as anticoagulant. The cells were washed and resuspended in either PBS (Dulbecco), or in plasma from the original blood sample. DNA was extracted using a kit (ZeptoMetrix Corp., Buffalo, NY, USA) that is based on the chaotropic extraction procedure. The DNA was denatured in aqueous solution containing desferal and enzymatically hydrolysed. Chaotropic extraction and the use of desferal are accepted methods for minimising artifactual oxidation of the DNA. Most oxidatively induced DNA base modifications, such as the formamide and thymine glycol modifications, inhibit hydrolysis by nuclease P1 of the phosphoester bond 3′ to the modified deoxyribonucleoside (Falcone and Box, 1997). Consequently, these lesions appear in a nuclease P1 (plus alkaline phosphatase) hydrolysate of DNA predominately as modified dinucleoside monophosphates; for example as in Figure 1A and B. Measuring these lesions at the dimer level has advantages: (1) The sensitivity of liquid chromatography-tandem mass spectrometry (LC-MS/MS) detection, using electrospray ionisation and operating in the negative ion mode, is better for dinucleoside monophosphates than for nucleosides (Bailey *et al*, 2006); (2) Isotopically labelled internal standards for quantifying these lesions can be obtained by labelling the nucleoside 3′ to the modified nucleoside. As the inhibition of nuclease P1 by the modified nucleoside is not absolute, it would seem that quantification at the dimer level is problematical. However, this difficulty is circumvented by adding the internal standard in the form of an oligomer bearing the lesion and the label to the DNA before digest. Internal standards (ZeptoMetrix Corp.) of the form d(GpApApT^f^pA^*^) and d(GpCpApT^g^pA^*^) were added to the denatured DNA sample before enzymatic digest where the asterisk indicates a purine base ^15^N labelled at all positions. As modified dinucleoside monophosphate derived from the DNA from the sample and from the internal standard are chemically identical, loss of product due to partial hydrolysis is accounted for in the final quantification step. The measurement of DNA damage at the dimer level has been described previously (Bailey *et al*, 2006; Greene *et al*, 2007). Samples contained 100 *μ*g of DNA. The samples were processed by HPLC before analysis by LC-MS/MS. The eluant between peaks due to unmodified deoxyribonucleosides was collected for analysis. The purpose of the HPLC step was to remove salt and unmodified nucleosides, thus reducing the amount of the sample injected into the mass spectrometer.

The effects of two forms of radiation commonly used to generate ROS, namely ionising radiation and UVC, were used to treat cells. The hydroxyl radical is the most significant ROS associated with ionising radiation, whereas the likely active agent in the case of UVC is singlet oxygen.

## Results

### DNA damage induced *in vitro*

**Figure 2:**
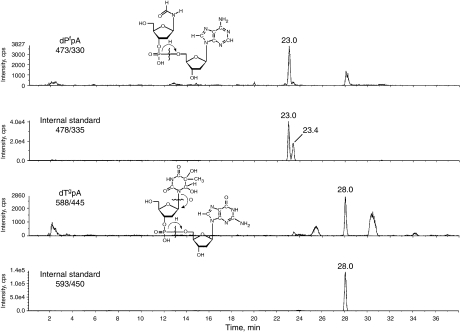
LC-MS/MS elution profiles of the formamide and thymine glycol modifications obtained from the DNA of untreated WBC from a healthy donor. The second trace in each example is from the internal standard, which serves to quantify the measurement.

*In vitro* experiments were carried out to compare background levels of DNA damage in WBC *vs* levels induced by familiar sources of ROS. Two modalities commonly used to generate ROS are ionising radiation and UVC. WBC resuspended in plasma from the original blood sample were subjected to ionising radiation (500 Gy). Levels of d(P^f^pA), Figure 1A, and d(T^g^pA), Figure 1B, were measured by LC-MS/MS. A typical set of LC-MS/MS elution profiles is shown in Figure 2. Quantitative results are presented in Table 1. A significant increase was observed for the thymine glycol lesion with little increase in the formamide lesion. The increase in d(T^g^pA) produced in WBC exposed to 500 Gy was found to be 3.46 fmol *μ*g^−1^ of DNA. Taking into account all four sequences of d(T^g^pN), where N stands for a normal nucleoside, this yield can be expressed as 3.55T^g^/10^6^N.

WBC were resuspended in PBS and exposed to 250 J m^−2^ of UVC. The results are included in Table 1. For this modality, formamide damage is more significantly enhanced than thymine glycol damage. The formamide modification has received relatively little attention as a product of oxidative stress, probably because it is not conveniently detected at the monomer level.

### DNA damage in WBC of ovarian cancer patients and controls

The levels of the formamide and thymine glycol modifications in ovarian cancer patients *vs* controls are compared in Table 2. The level on average of the formamide modification, d(P^f^pA), is higher in the cancer patients (6.38 fmol *μ*g^−1^) than in controls (5.56 fmol *μ*g^−1^). The level on average of the thymine glycol modification, d(T^g^pA), is also higher in the cancer patients (2.83 fmol *μ*g^−1^) than in controls (2.16 fmol *μ*g^−1^). The difference statistic between the mean values of the patient and control samples was analysed in terms of *t*-values. The *α*-values for the formamide and thymine glycol modification, based on the *t*-distribution for 32 degrees of freedom, are 0.25 and 0.15, respectively (van Belle *et al*, 2004.

**Figure 3:**
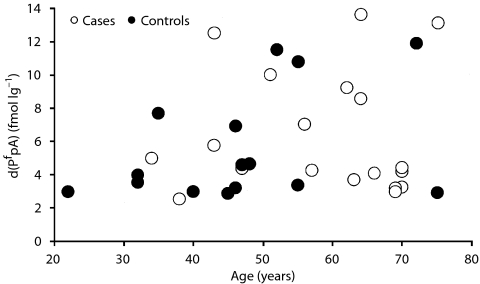
Range and distribution of ages of case and control participants plotted against d(P^f^pA) levels.

**Figure 4:**
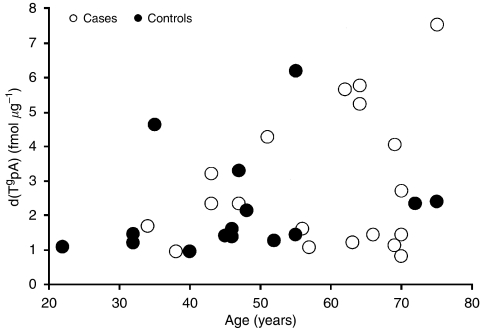
Range and distribution of ages of case and control participants plotted against d(T^g^pA) levels.

A principal concern in the interpretation of the results of Table 2 is a potential age factor. Figures 3 and 4 display the range and distribution of ages for the patient and control groups. The distributions are plotted *vs* the levels of d(P^f^pA) and d(T^g^pA), respectively. The age distributions for cases and controls are not identical. An *ad hoc*, but insightful, approach to evaluating age as a possible confounding factor. The control data to the equation *y*=*a*x+*b*, where *y* stands for the level of damage, d(P^f^pA), and *x* stands for the age of the individual in years. A least squares best fit of the d(P^f^pA) data as a function of age yielded values of 0.090 fmol *μ*g^−1^ per year and 1.343 fmol *μ*g^−1^ for *a* and *b*, respectively. These values were used to calculate the expected levels of d(P^f^pA) in cancer patients based on their individual ages. The mean value of d(P^f^pA) for the 19 cancer patients was calculated. The value of 5.57 fmol *μ*g^−1^ was obtained which is substantially the same value listed in Table 2. Thus, the relevance of the higher measured level of d(P^f^pA) in cancer patients compared with controls is of doubtful significance. On the other hand, the levels for d(T^g^pA) appear to be more meaningful. The least squares best fit for the control data of Figure 4 yielded values of 0.023 fmol *μ*g^−1^ per year and 1.081 fmol *μ*g^−1^. The calculated mean value of d(T^g^pA) in cancer patients was 2.16 fmol *μ*g^−1^ which is significantly below the measured value of 2.83 fmol *μ*g^−1^.

Other factors that may influence our results merit consideration. Berwick and Vineis (2000) reviewed 64 studies of cancer in relation to DNA damage and repair and tabulated the covariates considered in each study. Age, gender and smoking habits were the covariates most frequently considered. (The next most frequent practice in these studies was to omit consideration of cofactors altogether.) We have already considered the age factor and, of course, all of our study participants are women. An interesting insight came from background information on smoking habits. Of the 19 patients, 10 were former smokers. Only one reported being a current smoker (2 cigarettes per day). Similarly, 7 of 15 women in the control group were former smokers but none is a current smoker. Our conclusion is that the smoking habit is on the wane. Alcohol usage was similar in the two groups, 8 of 19 and 10 of 15 in cases and controls, respectively. Cancer incidence among immediate family members was also similar, 10 of 19 for cases and 8 of 15 for controls. For women taking birth control medications, the ratios were 8 of 19 and 9 of 15 in cases and controls, respectively.

Berwick and Vineis (2000) discuss whether an increase in DNA damage associated with cancer may be caused by the tumour itself, that is, reverse causation. For example, it is hypothesised that cancer cells release ROS into the blood stream. It seems unlikely, however, that any one reverse-causation mechanism would account for the increase in oxidative DNA damage observed in a wide range of malignancies (see next section). Bhatti *et al* (2008) have addressed the question of reverse causation experimentally. These investigators found no evidence of reverse causation using the comet assay to measure DNA damage in women before and after diagnosis.

## Discussion

The hypothesis that oxidative stress is a risk factor for cancer received considerable impetus with the realisation that oxidative stress probably originates primarily from normal metabolic processes (Ames *et al*, 1992). Cells synthesise ATP and in the process generate ROS, particularly hydrogen peroxide and superoxide. ROS-induced DNA damage generates mutations that presumably contribute to tumorigenesis. Thus, oxidative stress is a plausible global cause of cancer.

In this study two ROS-induced pyrimidine modifications of DNA were studied, namely the formamide remnant derived from pyrimidine bases and the thymine glycol modification of thymine base. Both modifications are produced in cells exposed to ionising radiation or to UVC (Bailey *et al*, 2006; Greene *et al*, 2007). However, the damage profiles are different depending on the ROS source, illustrating the advantage of evaluating oxidative stress through the simultaneous measurement of more than one lesion. LC-MS/MS technology permits the simultaneous measurement of multiple lesions (see below).

Evidences from many laboratories have supported the observation that oxidatively induced DNA damage, as measured by the level of 8-oxo-7,8-dihydroguanine in WBC of peripheral blood, on average, is higher in cancer patients (see Table 3). It is helpful to review the measurements of oxidative DNA damage in terms of percentage increases in the level of DNA damage relative to each laboratory's control value. In Table 3, we have taken the liberty of presenting data reported by other laboratories in a format somewhat different from the original reports. This is done to facilitate discussion and comparison with our own data. Table 3 lists the levels of the 8-oxo-7,8-dihydroguanine modification, dG^h^, measured in the DNA of WBC of cancer patients and in healthy controls. Typically, 8-oxo-7,8-dihydroguanine data, obtained by electrochemical or ^32^P-postlabeling methods, are reported in terms of dG^h^/10^6^dG where dG stands for deoxyguanine. In Table 3, the values are converted to dG^h^/10^6^N where N stands for normal bases. The consistent increase in the level of dG^h^ in cancer patients relative to healthy controls is strong evidence that cancer is related to oxidative stress. On the other hand, though measurements of dG^h^/10^6^N do distinguish between cancer groups and healthy control groups, the variation in dG^h^/10^6^N measured among control populations is disconcerting. The background/control levels reported in Table 3 differ by more than two orders of magnitude. Among sizeable healthy populations dG^h^/10^6^N should be a quasi-fixed quantity. The variation is usually ascribed to artifactual oxidation of guanine (Collins *et al*, 2004; ESCODD *et al*, 2005). An expedient is to accept each laboratory's determination of the background level according to its own method and calculate the percentage increase in the patient level. This has been done in Table 3. The percentage increases reported for all cancer types are fairly consistent.

Added to Table 3 are measurements from this study of the formamide and thymine glycol modifications in patients having ovarian cancer and in healthy female controls. For MS measurements it is customary to quantify products in terms of moles per weight of injected material, as in Table 2. For our present purpose results have been converted into terms of dT^g^/10^6^N and dP^f^/10^6^dN. It is interesting to note that the values for dP^f^/10^6^N and dT^g^/10^6^N fall close to the median values reported for dG^h^/10^6^N.

The levels of the formamide and thymine glycol lesions are lower in our control group compared with the patient group. However, as noted above, it is problematical whether the increase in the formamide modification is related to cancer. Nevertheless, the trend is the same for the pyrimidine modifications as for 8-oxo-7,8-dihydroguanine and our results add weight to the hypothesis that oxidative DNA damage is a contributor to the multiple mutations occurring during tumorigenesis (Sjoblom *et al*, 2007; Wood *et al*, 2007.

It is unlikely that measurements of the formamide and thymine glycol base modifications will be plagued by the difficulties that have attended the measurements of 8-oxo-7,8-dihydroguanine (Collins *et al*, 2004; ESCODD *et al*, 2005). This expectation is based on the lower ionisation energy of the pyrimidine bases and less likely artifactual oxidation of pyrimidine bases compared with guanine (Halliwell, 2000; Steenken *et al*, 2000).

It is feasible to measure multiple DNA modifications simultaneously using the LC-MS/MS technology. In this connection an additional remark may be made concerning our practice of measuring DNA damage at the dimer level. The selectivity, sensitivity and the ability to measure multiple modifications simultaneously makes LC-MS/MS technology well suited for assessing DNA damage. But to realise these advantages one must know beforehand the molecular weights of the product of interest and of a principal fragmentation product. By assaying at the dinucleoside monophosphate level a predictable fragment is always obtained from the 3′ end of the molecule independent of the nature of the modified 5′ end. Thus, the second MS value can be programmed for one of four values. It becomes feasible to perform a comprehensive survey of a DNA digest by programming the first MS value over a range of mass values. Using this approach we have observed signals in the DNA from WBC due to five modifications, all of which are more abundant than either 8-oxo-7,8-dihydroguanine or the thymine glycol lesions. We hope to use this broader spectrum of DNA modifications to better define the relationship between oxidative stress and cancer risk (Wang *et al*, submitted) and to clarify the role of antioxidants in the prevention of cancer. Molecular biomarkers may provide clearer guidance on the use of antioxidants in the prevention of cancer than is currently available from epidemiology studies (Hercberg *et al*, 2006; Reid *et al*, 2008; Sesso *et al*, 2009).

## Figures and Tables

**Table 1:** Levels of formamide and thymine glycol lesions in WBC compared with levels in WBC exposed to 500 Gy X-ray or 250 J m^−2^ UVC

	**dP^f^pA *μ*g^−1^**	**dT^g^pA *μ*g^−1^**
Samples (5), control	4.89±0.76	2.23±0.34
Samples (5), 500 Gy	5.37±1 02	5.69±1.02
Samples (8), control	5.76±0.75	4.48±1.01
Samples (8), 250 J m^−2^	14.6±2.08	8.41±1.36

Values are given in femtomoles of lesion per microgram of DNA.

Numbers in parentheses indicate the number of donors; error measures are standard errors of the mean.

**Table 2:** Levels of the formamide and thymine glycol modifications in WBC of ovarian cancer patients and healthy controls

	**dP^f^pA *μ*g^−1^**	**dT^g^pA *μ*g^−1^**
Ovarian cancer patients (19)	6.38±0.83	2.83±0.45
Controls (16)	5.56±0.86	2.16±0.38

Values are given in femtomoles of lesion per microgram of DNA.

Numbers in parentheses indicate the number of donors; error measures are standard errors of the mean.

**Table 3:** Oxidative DNA damage in cancer and control groups

**Study group**	**Control (*n*)**	**Study group (*n*)**	**Δ%**	**Reference**
Lung cancer, dG^h^/10^6^N	7.30±1.12 (48)	11.6±1.54 (46)	+59	Vulimiri *et al* (2000)
Leukemia, dG^h^/10^6^N	8.52±0.55 (11)	13.6±0.72 (22)	+60	Oltra *et al* (2001)
Colorectal cancer, dG^h^/10^6^N	1.91±0.13 (35)	2.74±0.23 (36)	+43	Gackowski *et al* (2002)
Bladder cancer, dG^h^/10^6^N	4.36±0.19 (22)	7.52±0.58 (29)	+71	Akcay *et al* (2003)
Breast cancer, dG^h^/10^6^N	121±18 (32)	278±29 (29)	+128	Soliman *et al* (2004)
Oesophagial cancer, dG^h^/10^6^N	0.98±0.06 (43)	1.44±0.12 (17)	+46	Breton *et al* (2005)
Ovarian cancer, dP^f^/10^6^N	5.63±0.87(15)	6.43±0.84(19)	+14	This report
Ovarian cancer, dT^g^/10^6^N	2.16±0.38(15)	2.83±0.45(19)	+31	This report

Measurements of 8-oxo-7,8-dihydroguanine , formamide and thymine glycol modifications in cancer and control groups.

Values are dG^h^/10^6^N, dP^f^/10^6^N or dT^g^/10^6^N±s.e.m.; numbers in parentheses indicate the number of participants.
